# Does cutting down on your food consumption lead to a net improvement in nutritional intake? A panel data approach using data from the UK Biobank

**DOI:** 10.1186/s12889-023-17217-y

**Published:** 2023-11-17

**Authors:** Luke B. Wilson, Robert Pryce, Esther C. Moore, Lucy Burke, Penny Breeze

**Affiliations:** School of Medicine and Population Health, University of Sheffield, Sheffield, UK

**Keywords:** Food, Diet, HFSS, Food Substitution

## Abstract

**Background:**

Food diets are complex and a policy targeting one item of a person’s diet does not affect their nutritional intake in a solely additive or subtractive manner. Policies tackling unhealthy diets are more likely to be adopted by governments if there is robust evidence to support them. To evaluate dietary policies, it is important to understand the correlations and interdependencies between food groups, as these can lead to unintended negative consequences. We aimed to see whether reductions in consumption of a particular group is related to a net improvement in nutritional intake, after taking into account patterns of consumption and substitution across food groups.

**Methods:**

Detailed dietary data was collected using a 24-h online dietary assessment from the UK Biobank and Oxford Web Q (*n* = 185,611). We used panel data fixed effects methods to estimate changes in energy, saturated fat, total sugar, and fibre following a 100gram reduction across 44 food groups. We compare these estimates against the average nutritional value of that food group from the UK National Diet and Nutrition Survey.

**Results:**

We find evidence of variation in whether a food is compensated between the main confectionery products. Crisps, savoury snacks, and sugar confectionery are less likely to be compensated, whereas chocolate confectionery, biscuits, and buns/cakes/pastries and pies are compensated. The result is particularly striking for chocolate confectionery which shows that while chocolate confectionery often has a high energy content, eating less chocolate confectionery is not associated with an equal reduction in energy. Instead, we find individuals switch or compensate for their reduction in chocolate confectionery consumption with other high energy food items.

**Conclusions:**

We find that sugar confectionery and crisps and savoury snacks are less likely to result in substitution than chocolate confectionery. This would suggest that food policies aiming to reduce the consumption of these food groups are more likely to result in overall lower consumption of unhealthy foods.

**Supplementary Information:**

The online version contains supplementary material available at 10.1186/s12889-023-17217-y.

## Introduction

In the United Kingdom (UK) 26.2% of adults are obese and a further 36% are classified as overweight, which equates to one of the highest rates of obesity in Western Europe [1]. As a result, people in the UK live on average 2.7 years less due to being overweight compared to those in the healthy weight range and being overweight accounts for 8.4% of health expenditure [2].

The impact of obesity on associated chronic diseases has led to calls for a comprehensive public health approach to tackling the current trends in physical inactivity and high energy diets. In response, food has attracted the attention of policymakers in the UK, who have typically been aiming to change the behaviours and consumption patterns of the public towards healthier options through a variety of different channels. This has led to price based policies such as a levy placed on all sugar sweetened beverages with more than 5 g of sugar per 100ml^1^ (introduced in April 2018), and a proposed ban on price promotions of food high in fat, sugar and salt (HFSS) [3]. There has also been the introduction of non-pricing policies and initiatives; such as bans on advertisements of foods which are HFSS, calorie labelling on out of home purchases [4], traffic light labelling, and voluntary reformulation [5]. Both price and non-price-based policies have a growing body of evidence to suggest their effectiveness at reducing the growing burden of obesity [6–10]. Non-price-based policies are often population-based policies that operate by nudging people to change habits rather than more explicit policies such as banning unhealthy foods. Evidence has shown that nudge strategies are effective in nutrition policy [11].

However, diets are complex and a policy targeting one item of a person’s diet does not affect their nutritional intake in a solely additive or subtractive manner. This is because food consumption patterns are correlated: if a person can be steered away from consuming a burger, it is also likely they will not eat the fries that typically accompany it. This would lead to a bigger health gain than just the reduction of (for example) fat that is in the burger. Conversely, if a policy steered a consumer away from eating a chocolate bar, they may substitute this with a sugary drink or other snack. In this example, this would lead to a smaller health gain than the reduction of (for example) the sugar in the chocolate bar.

Policies are more likely to be adopted by governments if there is robust evidence to support them. However, public health dietary policies are challenging to evaluate with prospective research methods. In order to evaluate dietary policies, it is important to understand the correlations and interdependencies between food groups, as these can lead to unintended negative consequences. For example, policymakers would benefit from knowing the effect of a ban on chocolate bars at supermarket checkouts on calorie intake. To do this – along with estimates of the effect of the policy on chocolate consumption – estimates of the associated changes in diet from reducing chocolate consumption are key. It is difficult to evaluate these types of policies with traditional randomised controlled trials and natural experiments using routine data are expensive and challenging to isolate the effect size. Assuming a simple relationship between the number of chocolate bars sold and the corresponding calorie intake, could be misleading. Whilst price-based policies can inform the estimates to some extent, price crucially changes the incentives to purchase certain goods. To model non-price policies, it is therefore important to understand changes in patterns of consumption without price incentives changing.

Previous evidence on the effect of non-pricing policies from the UK simulated the impact of an advertising ban of potato chips. They found that banning advertising and holding prices fixed, lowered potato chip demand, as well as total purchases of potato chip calories, saturated fat and salt. However, the authors found that these health gains were partially offset for two reasons. Firstly, they found that potato chip manufacturers responded to the ban by lowering prices, this led to an offsetting increase in potato chip demand. Secondly, some consumers switched out of the potato chip market and substituted their consumption to other, less healthy, junk foods [12]. Substitution effects have also been observed from studies changing energy density of food [13]. Understanding the overall nutrient impact of changes in demand may help target policies to foods with healthier substitution effects. Anticipating unintended consequences would provide opportunities to improve the design and implementation of policies to mitigate unhealthy substitution effects, for example by combining policies to produce synergistic effects [14].

This paper extends the literature by estimating the correlation of consumption of food types to help understand the effects of changing food consumption through non-price policies. We take advantage of a longitudinal dataset conducted in the United Kingdom (UK) to observe food patterns at multiple time-points. This repeated measure design removes some of the bias often associated with cross-sectional designs that do not account for confounding factors that would explain some variation in dietary patterns across individuals, such as unobservable differences like preferences in taste or particular food combinations. We calculate the change in total nutritional intake of an individual given a 100 g gram reduction in consumption across 44 food groups and compare this with the average nutritional value of that food group.

The aim of this paper is to see whether reductions in consumption of a particular group is related to a net improvement in nutritional intake, after taking into account patterns of consumption and substitution across food groups.

## Methods

### Data

This study uses data from the UK Biobank. UK Biobank is a population-based longitudinal study that recruited roughly 500,000 participants aged between 40 and 69 when they joined UK Biobank (from 2006 to 2010). It follows the health and wellbeing of the participants over a 17-year period with future data releases scheduled. The volunteer participants of UK Biobank completed a full baseline assessment, including self-reported measurements via touch-screen questionnaires as well as a verbal interview collecting a wide range of information on socio-demographic factors, lifestyle, and behaviours (i.e., history of smoking and sleep duration), and medical history. Physical measurements (i.e., height, weight, spirometry, blood pressure, heel bone density), blood and urine samples were also taken.

UK Biobank protocols and study details can be found on the UK Biobank website (https://www.ukbiobank.ac.uk/). UK Biobank is not representative of the general population with evidence of a ‘healthy volunteer’ selection bias, details of which are available online on the UK Biobank website (http://www.ukbiobank.ac.uk/wp-content/uploads/2011/11/UK-Biobank-Protocol.pdf).

We take advantage of a longitudinal dataset conducted in the UK by using repeated measures on an individual to control for time invariant unobserved heterogeneity and therefore reduce the bias often associated with studies that use a cross-sectional design. Whilst results are generated for all food types and nutrients, the paper presents selected food groups for illustrative purposes. Additional nutrient types are presented in the supplementary materials.

### Dietary assessment

Data on dietary intake were collected by all UK Biobank participants who provided a valid email address at recruitment. Participants were invited to complete the 24-hour online dietary assessment (Oxford WebQ), which is a web-based 24-h dietary assessment tool developed and evaluated for use in large population studies (www.ceu.ox.ac.uk/research/oxford-webq). The Oxford WebQ was collected toward the end of the baseline recruitment period of UK Biobank (2009–2010). Follow ups were conducted on up to four separate occasions (February 2011 to April 2011; cycle 2: June 2011 to September 2011; cycle 3: October 2011 to December 2011; cycle 4: April 2012 to June 2012) [15]. For the purpose of this study, we focus on respondents in the UK Biobank who have completed at least one Oxford WebQ. Our sample size is therefore 185,611 individuals.

The Oxford WebQ presents participants with 21 broad food groups, expanding to offer 206 commonly consumed food and 32 types of drinks. The participants are prompted to select the number of portions consumed over the previous 24 h, mostly from predefined categories offered to them (www.ceu.ox.ac.uk/research/oxford-webq). Until recently, the food composition table (FCT) and portion size used for the Oxford WebQ has been the UK McCance and Widdowson’s “The Composition of Foods 6th edition (2002). This has now been replaced by the UK Nutrient Databank (UKNDB) (2013), which provides food composition data measured closer in time to when participants completed the questionnaire in UK Biobank (2009–2012).

In the Oxford WebQ, nutrients are automatically estimated via built-in algorithms and food composition data [16]. For the purpose of this study, we focus on total energy intake, total fat, saturated fatty acids (SFA), monounsaturated fatty acids (MUFA), polyunsaturated fatty acids (PUFA), carbohydrates, total sugars, and fibre. The Oxford WebQ has been validated against biomarkers [17] and compared to interviewer-administered 24 h recalls [18] and showed acceptable reproducibility when using at least 2 dietary assessments [19, 20].

### Study design

Following the previous literature, we removed participants with implausible energy intakes [21]. These were defined as (men: < 3347 or > 17,573 kJ/days or < 800 or > 4200 kcal/days); women: < 2092 or > 14,644 kJ/days or < 600 or > 3500 kcal/days). Recorded food and drinks from the Oxford WebQ were classified into 44 groups according to their nutrient profile and the classification used in the UK National Diet and Nutrition Survey (NDNS). This allows us to make comparisons between the change in nutrient intake and the average nutrient intake of that food group in the NDNS. For the purpose of this study, we use data from Wave 11 of the NDNS 2018/19.

As mentioned previously, reductions in consumption of one food group may be correlated with reductions or increases in consumption of other food groups. To estimate the effect of a reduction in one food group on overall nutrient intake we use a fixed effects panel model for each food group and nutrient separately. That is, formally:

$${I}_{int} = \beta {C}_{ift}+ {\delta }_{i}+{\delta }_{t}+{\epsilon }_{ift}$$

where ${I}_{int}$ is intake by individual *i* of nutrient *n* at time period *t*, ${C}_{ift}$ is consumption by individual *i* of food group *f* at time *t*. ${\delta }_{i}$ is a fixed effect for individual *i* which captures individual heterogeneity and ${\delta }_{t}$ is a time fixed effect which captures changes over time that are applicable to everyone. ${\epsilon }_{ift}$ is a random error term. The $\beta$ coefficient is thus the estimate of the effect of a change in consumption of food group f on intake of nutrient n. The fixed effects model has particular strengths over a random effects model because it removes any bias from food preferences. For example, it may be that people who eat more chocolate also just have a taste for sugary foods generally. A model which didn’t control for individuals’ preferences would see a positive relationship between the number of chocolate bars eaten and sugar consumption, whereas at the individual level people may also be less likely to drink a sugary drink when they eat a chocolate bar and so the relationship is not as strong.

Utilising data from wave 11 of the NDNS food level diary data, we calculate the average nutritional content of foods within each of the 44 food groups. This allows us to compare our estimated change in nutrient intake against the average nutritional value of that particular food group consumed in wave 11.

## Results

### Summary statistics

Table 1 depicts the summary statistics of the UK Biobank. The average age of a respondent in the UK Biobank is 58.83 years old. The UK Biobank sampled individuals aged 40 to 69 from 2006 to 2010 therefore this is reflected in the average age. In our sample, there are more females 55%.

**Table 1:** Summary Statistics from the UK Biobank

	Mean (%)	Standard Deviation	Min	Max
**Socio-Demographics**				
Age	58.83	7.85	40	74
*Sex*				
Male	83, 530 (45.00)			
Female	102,081 (55.00)			
**Nutrition Groups**				
Energy (Kcal)	2052.44	597.20	500.45	4199.23
Protein (g)	81.10	26.16	2.15	364.07
Carbohydrates (g)	249.71	83.14	0.00	771.09
Saturated Fats (g)	28.83	13.25	0.00	126.14
Polyunsaturated Fats (g)	13.95	8.02	0.00	74.11
Monounsaturated Fats (g)	32.68	14.03	0.00	147.12
fat	75.46	31.21	0.00	265.72
Total Sugars (g)	119.25	50.04	0.00	585.68
Fibre (g)	16.73	7.08	0.00	98.53
**N**	185,611			

### Estimated change in nutritional intake

Each figure illustrates the difference between the average nutritional value of a particular food group and the estimated individual change in nutrient intake if consumption were to fall by 100 g.

The estimated reduction in nutrient intake is calculated from the UK Biobank. This illustrates the change in nutrient intake made by respondents if they were to reduce their consumption of a particular food group by 100 g. The average nutrient intake per 100 g is the average nutritional value of that particular food group calculated from the NDNS.

If the estimated reduction in nutrient intake is lower than the average nutritional value group this means that individuals have reduced their intake more than the average value of that food group. For example, this means that if an individual eats 100 g less of chocolate a day and this corresponds to 505.6 kcal from the NDNS data, if the estimated reduction in nutrient intake from the UK Biobank data is more than this the participant has not only cut this chocolate bar from their diet but also other foods that are also high in energy.

In contrast, if the average nutrient intake is higher than the estimated reduction in nutrient intake in absolute terms this means that while an individual has reduced their consumption of that food group, they have substituted that with an alternative food item that is also high in that particular nutrient. Going back to the chocolate bar example, eating 100 g less chocolate would remove approximately 505.6 kcal from an individual’s diet based on the average nutritional content of chocolate. Whereas the model estimates that total daily calorie intake is actually reduced by 132.3 kcal, when chocolate consumption is reduced by 100 g. Therefore, individuals are compensating for their reduction in chocolate and consuming other products to replace the energy not in the chocolate confectionery category.

**Fig. 1:**
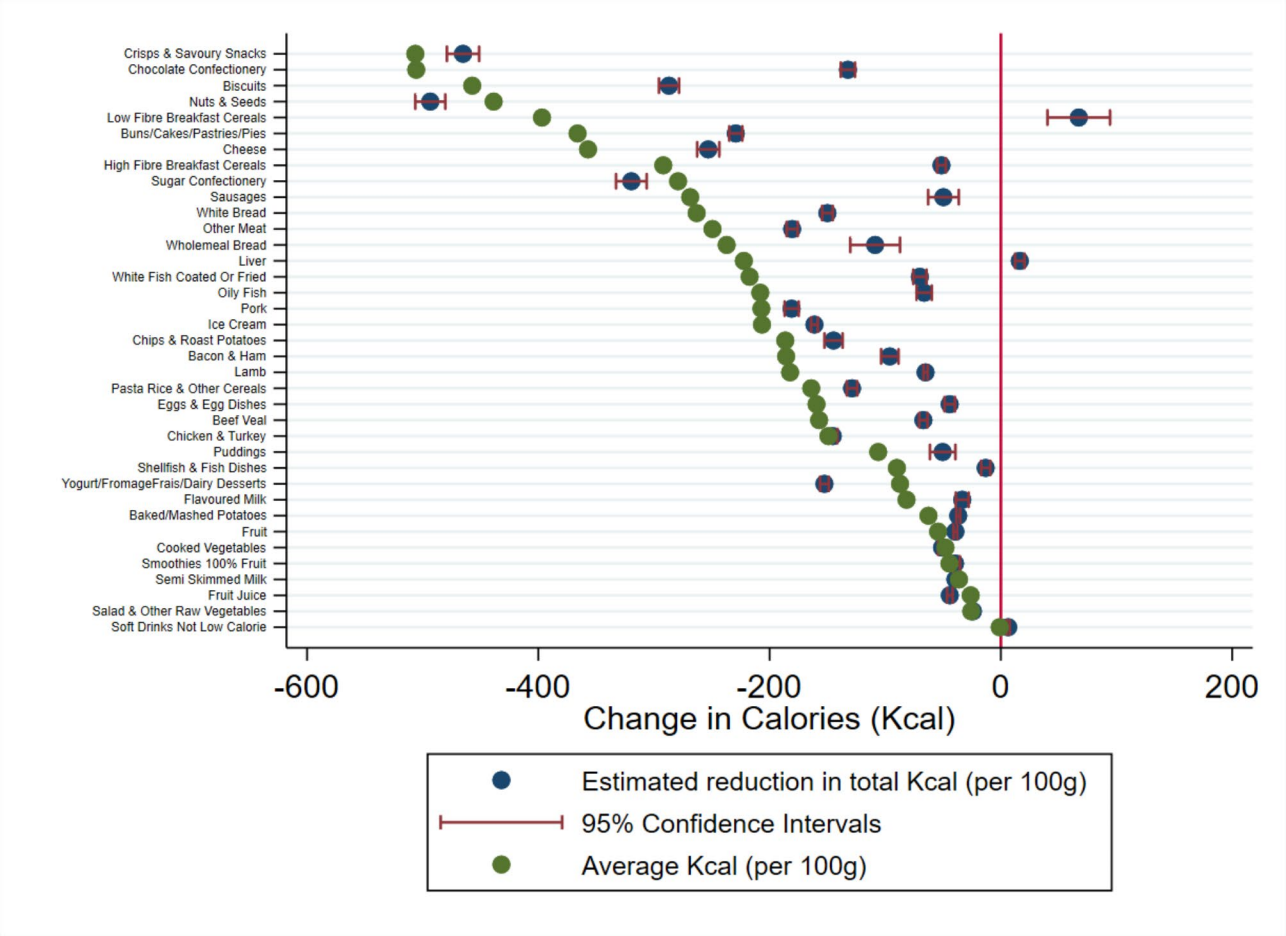
Estimated change in daily energy consumed following a reduction in consumption of that food group The blue dot illustrates the change in daily energy consumption estimated form the UK Biobank data (N = 185,611) with precision illustrated by the red confidence intervals. The green dot illustrates the average energy content (kcal) for 100 g of each food group. The red line at zero illustrates a point where no change in energy is observed for a 100 g reduction in the consumption of each food group. Kcal kilocalories

Figure 1 illustrates the estimated reduction in total energy consumed by participants in the UK Biobank if they consumed 100 g less of that food group. From this figure, it is evident that there is a lot of variation in whether a food is compensated between products. Crisps and savoury snacks as well as sugar confectionery are less likely to be compensated, which all illustrate small gaps between the estimated reduction in total energy, whereas chocolate confectionery, biscuits, and buns/cakes/pastries and pies are compensated. The result is particularly striking for chocolate confectionery which shows that while chocolate confectionery often has a high calorie content, eating less chocolate confectionery is not associated with an equal reduction in energy. Instead, we find individuals switch or compensate for their reduction in chocolate confectionery consumption with an almost equal consumption of other foods.

There are differences in the magnitude and direction of the compensation for ice-cream, yoghurt-based products, and puddings. We find that ice cream and puddings are compensated whereas for yoghurts the estimated reduction in energy is higher than the average energy per 100 g. In addition, we find that reducing consumption of both high and low fibre breakfast cereals leads to compensation of other calorific food items. Particularly in the case of low fibre breakfast cereals, we find that individuals in fact increase their calorie intake if they reduce their consumption of cereal.

**Fig. 2:**
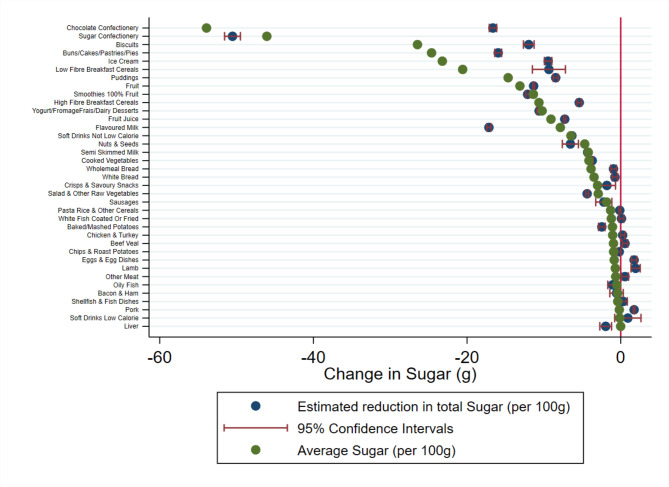
Estimated change in daily total sugar consumed following a reduction in consumption of that food group The blue dot illustrates the change in daily sugar (gram) consumption estimated form the UK Biobank data (N = 185,611) with precision illustrated by the red confidence intervals. The green dot illustrates the average sugar content (g) for 100 g of each food group. The red line at zero illustrates a point where no change in sugar is observed for a 100 g reduction in the consumption of each food group. g grams

**Fig. 3:**
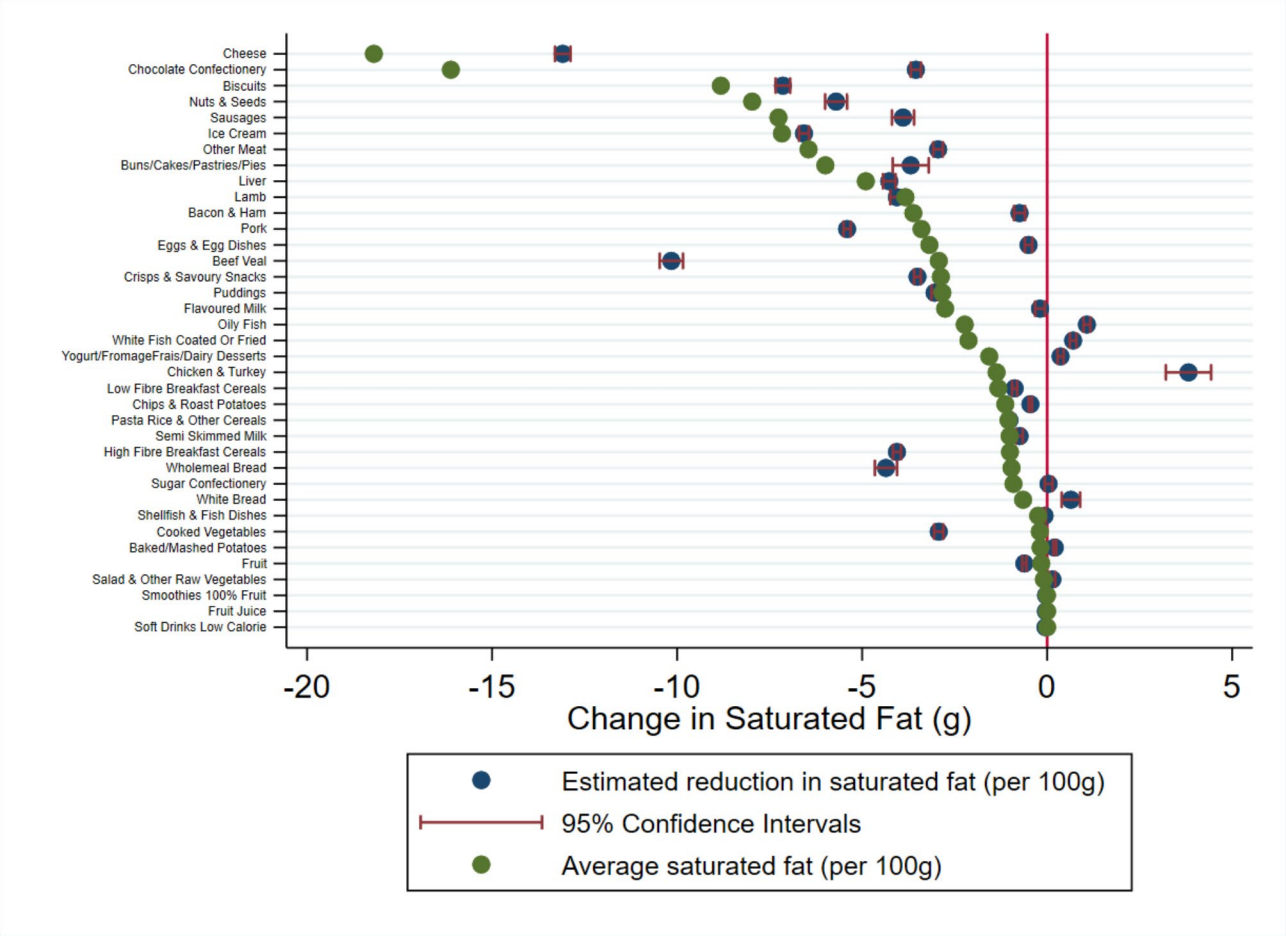
Estimated change in daily saturated fat consumed following a reduction in consumption of that food group The blue dot illustrates the change in daily saturated fat (g) consumption estimated form the UK Biobank data (N = 185,611) with precision illustrated by the red confidence intervals. The green dot illustrates the average saturated fat (g) content for 100 g of each food group. The red line at zero illustrates a point where no change in saturated fat is observed for a 100 g reduction in the consumption of each food group. g grams

In Fig. 2 we present our findings for total sugar intake. We find similar results as for energy. Again, we find that sugar confectionery is not compensated for, whereas most other high sugar foods do have some compensation, to varying degrees. Chocolate confectionery is highly compensated for in terms of total sugar intake. Figure 3 depicts the change in saturated fat intake. Among HFSS products, our results are consistent with our previous measures of nutrient intake. We present similar findings for chocolate confectionery and crisps and savoury snacks. Individuals are more likely to consume less saturated fat if they were to cut down on crisps and savoury snacks. In contrast, individuals are more likely to compensate for their reduction of chocolate and biscuits consumption.

However, we do find interesting patterns for the various meat types. Reducing consumption of beef/veal, pork, and lamb lead to the consumption of less saturated fat, while sausages and bacon lead to partial substitution, and chicken and fish lead to the consumption of more saturated fat. Reducing consumption of low-fat proteins, such as chicken and fish, appears to be correlated with increased consumption of saturated fat. This is because these are substituted for fattier alternatives. Whereas, beef and pork are perhaps high fat (depending on the cut of the meat), so will be substituted for lower fat alternatives. We present the results for other types of fats in the appendix.

**Fig. 4:**
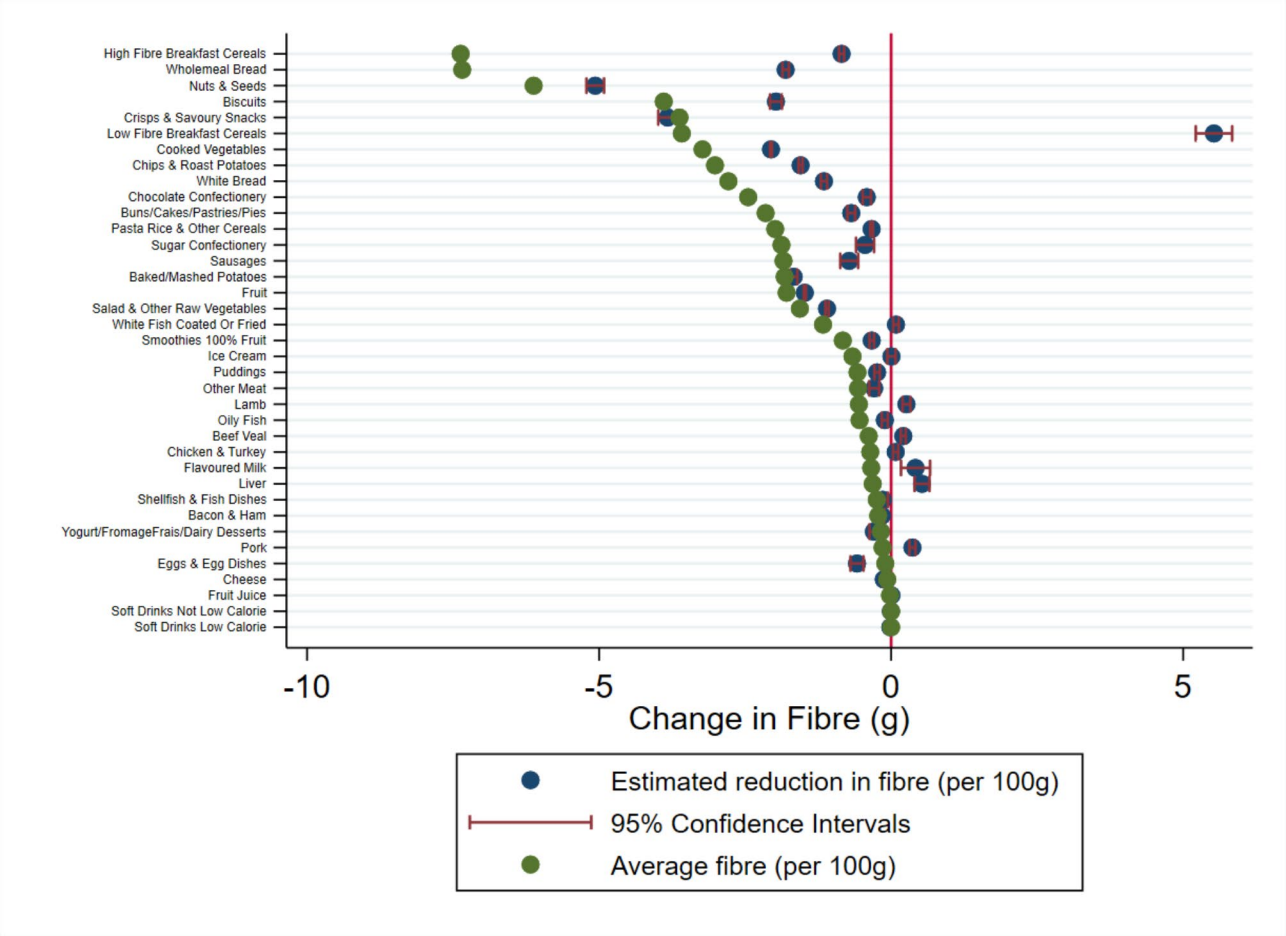
Estimated change in daily fibre consumed following a reduction in consumption of that food group The blue dot illustrates the change in daily fibre (g) consumption estimated form the UK Biobank data (N = 185,611) with precision illustrated by the red confidence intervals. The green dot illustrates the average fibre (g) content for 100 g of each food group. The red line at zero illustrates a point where no change in fibre is observed for a 100 g reduction in the consumption of each food group. g grams

Figure 4 illustrates the estimated reduction in total fibre consumed by participants in the UK Biobank if they consumed 100 g less of that food group. We find that cutting out high fibre breakfast cereal and wholemeal bread does not impact fibre intake. Instead, we find that people compensate for this loss of fibre by eating other fibre rich food items. In contrast, individuals do not compensate for loss of fibre from consuming fruit and vegetables so maintain a key part of a balanced healthy diet. Whereas a good way of getting people to eat fibre is to take away low fibre breakfast cereal.

## Discussion

Our study provides a deeper understanding of how individuals compensate their nutrient intake when they cut down/reduce their consumption of various types of food. We examine whether an individual substitutes their consumption of a particular food group with other foods which impacts on overall nutritional intake. This paper focuses on four main nutritional values, energy, total sugar, saturated fat, and fibre.

We use panel data from the UK Biobank to estimate the effect of a reduction in disaggregated food groups on overall nutrient intake, controlling for individual heterogeneity. We corroborate this data from the NDNS to provide comparisons between an individual’s estimated reduction in nutrient intake and the average nutritional value of that product. We find that while chocolate confectionery often has a high calorie content, eating less chocolate confectionery is not associated with an equal reduction in energy. Instead, individuals switch or compensate for their reduction in chocolate confectionery consumption with other high calorie food items. This result is consistent with other HFSS foods such as biscuits and low fibre breakfast cereals. This result is also consistent across other nutritional measures such as total sugars and saturated fats. In addition, we find that sugar confectionery and crisps have much smaller substitution effects across all the estimated nutrition measures, meaning that individuals are less likely to swap their sugar/crisp consumption for a similar food product.

Previous evidence in the UK found that sweet snacks were more price sensitive than savoury snacks and substitution to other foods would further reduce energy intake [22]. In a simulation study a 20% price rise for sweet snacks translated into an approximately 45 kcal reduction in energy per person per day on average, of which 11 kcals were attributable to substitution effects. A 20% price rise for savoury snacks would lead to a 15-kcal reduction of which approximately 4 kcals were attributable to savoury snacks. In our analysis we find that reducing the consumption of chocolate confectionery and biscuits is associated with small decreases in energy intake. However, we find a larger reduction in energy associated with a change in crisps and savoury snack consumption. Direct comparisons to this literature are difficult as the previous analysis focus on price-based responses whereas the mechanism for the reduction in sweet snacks is not controlled for in our analysis. However, the differences indicate that patterns in consumption behaviour and substitution effects may vary in the absence of price changes, but more research is needed to investigate this further.

Similar to the results found in our study, evidence from the UK evaluated the impact of a real-world intervention to remove seasonal promotions of chocolate confectionery. The policy had a large and significant effect on sales volume in a major UK supermarket. In contrast the effect on sugar was not significant and the effect on energy consumption was small and significant [19]. This provides evidence that nutritional benefits in real-world interventions can be difficult to observe and may be mitigated by substitution effects. In contrast modelling studies estimating the population health impact of public health policies often assume optimistic assumptions that unhealthy foods are not substituted or substituted with a healthy alternative, which may overestimate the benefits [23]. The estimates reported in this study provide a more robust prediction of the population-level substitution effects.

While we provide new evidence on food substitution and compensation our work is not without limitations. While we are able to take advantage of a longitudinal data source and track the consumption of individuals over time, the participants in the UK biobank are aged 40+. We are therefore unable to observe how younger individuals or children compensate for their food consumption. In addition, the UK Biobank is not representative of the general population with evidence of a ‘healthy volunteer’ selection bias. Finally, the UK Biobank does not estimate daily sodium intake from the Web-Q questionnaire. We are therefore unable to estimate sodium intake, and how this may change following food compensation.

The availability of substitute foods (eaten instead) and complementary foods (eaten together) are critical factors that determine what food gets eaten. This creates a challenging environment to implement population-level public health policies to modify diet, because our appetites and preferences for calorie-dense foods will override good intentions and result in consumption of substitute goods.

## Conclusion

Our analysis identifies variation in the effects on nutrition of alternative food types. We find that sugar confectionery and crisps and savoury snacks are less likely to result in substitution than chocolate confectionery. This would suggest that food policies aiming to reduce the consumption of these food groups could lead to less unintended substitution to other unhealthy foods.

### Electronic supplementary material

Below is the link to the electronic supplementary material.

Supplementary Material 1

## Data Availability

The data that support the findings of this study are available from UK Biobank and National Diet and Nutrition Survey Years 1–11, but restrictions apply to the availability of these data, which were used under license for the current study, and so are not publicly available. Data are however available from the data holders (UK Biobank and UKDS) upon reasonable request and with permission of UK Biobank and UKDS. https://www.ukbiobank.ac.uk/. https://ukdataservice.ac.uk/.

## Footnotes

1 The sugar sweetened beverage tax in the UK is as follows: £0.24 per litre of drink if it contains 8 g of sugar per 100 millilitres and £0.18 per litre of drink if it contains between 5 and 8 g of sugar per 100 millilitres.
