# Supplementary material for: Does cutting down on your food consumption lead to a net improvement in nutritional intake? A panel data approach using data from the UK Biobank
Source: BMC Public Health. 2023 Nov 17;23:2274. doi: 10.1186/s12889-023-17217-y (PMC10656843; doi:10.1186/s12889-023-17217-y)
Supplement: Supplementary file 1 — Supplementary Material 1 [file 12889_2023_17217_MOESM1_ESM.docx]

**Does cutting down on your food consumption lead to a net improvement in nutritional intake? A panel data approach using data from the UK Biobank**

**Supplementary Materials**

Luke B. Wilson^1^

Rob E. Pryce^1^

Esther C. Moore^1^

Lucy Burke^1^

Penny Breeze^1^

^1^ School of Health and Related Research, University of Sheffield

^*^ Corresponding author. Address: School of Health and Related Research, University of Sheffield Email: [l.b.wilson@sheffield.ac.uk](mailto:l.b.wilson@sheffield.ac.uk)

Figure A1: Estimated change in daily poly unsaturated fat consumed following a reduction in consumption of that food group
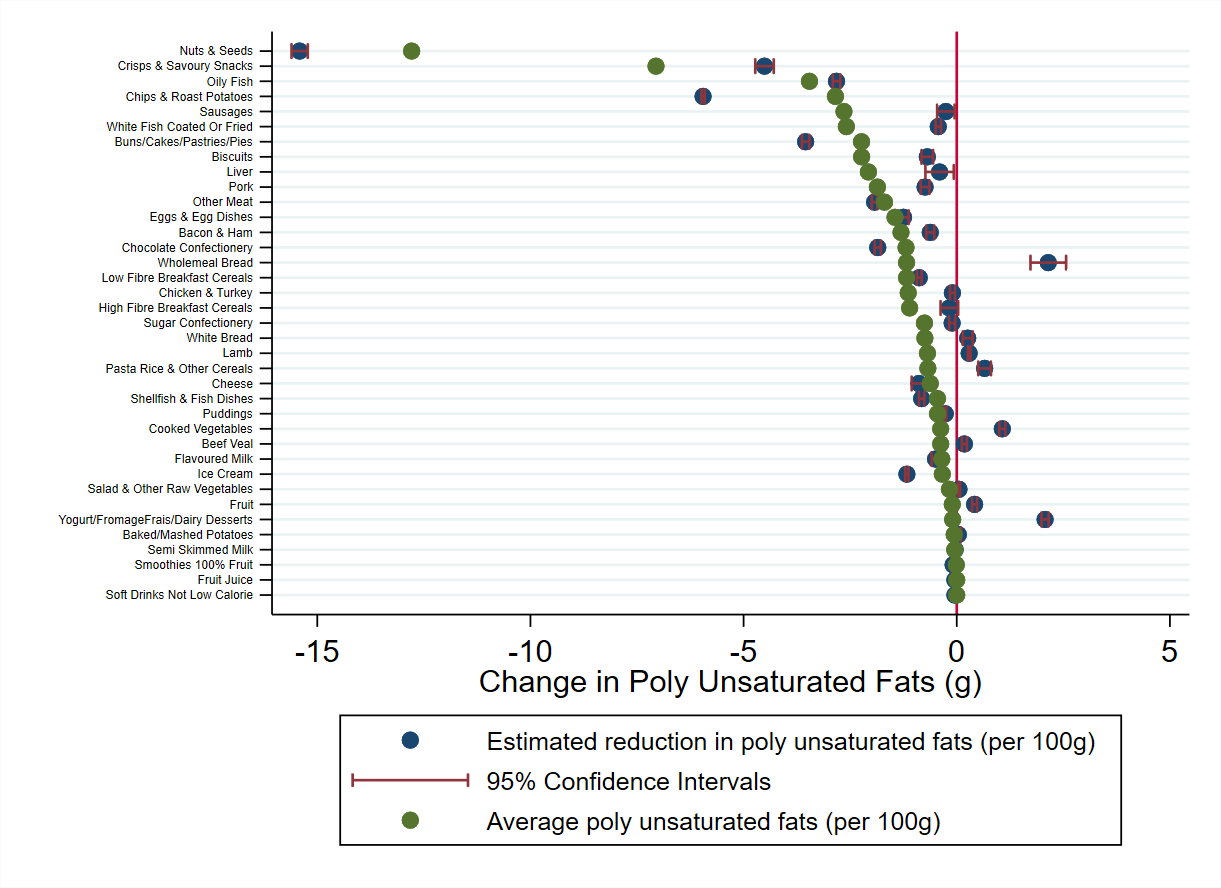


Figure A1 legend: The blue dot illustrates the change in daily poly unsaturated fat consumption estimated form the UK Biobank data (N=185,611) with precision illustrated by the red confidence intervals. The green dot illustrates the average poly unsaturated fat for 100g of each food group. The red line at zero illustrates a point where no change in poly unsaturated fats is observed for a 100g reduction in the consumption of each food group. g grams.

Figure A2: Estimated change in daily carbohydrates consumed following a reduction in consumption of that food group


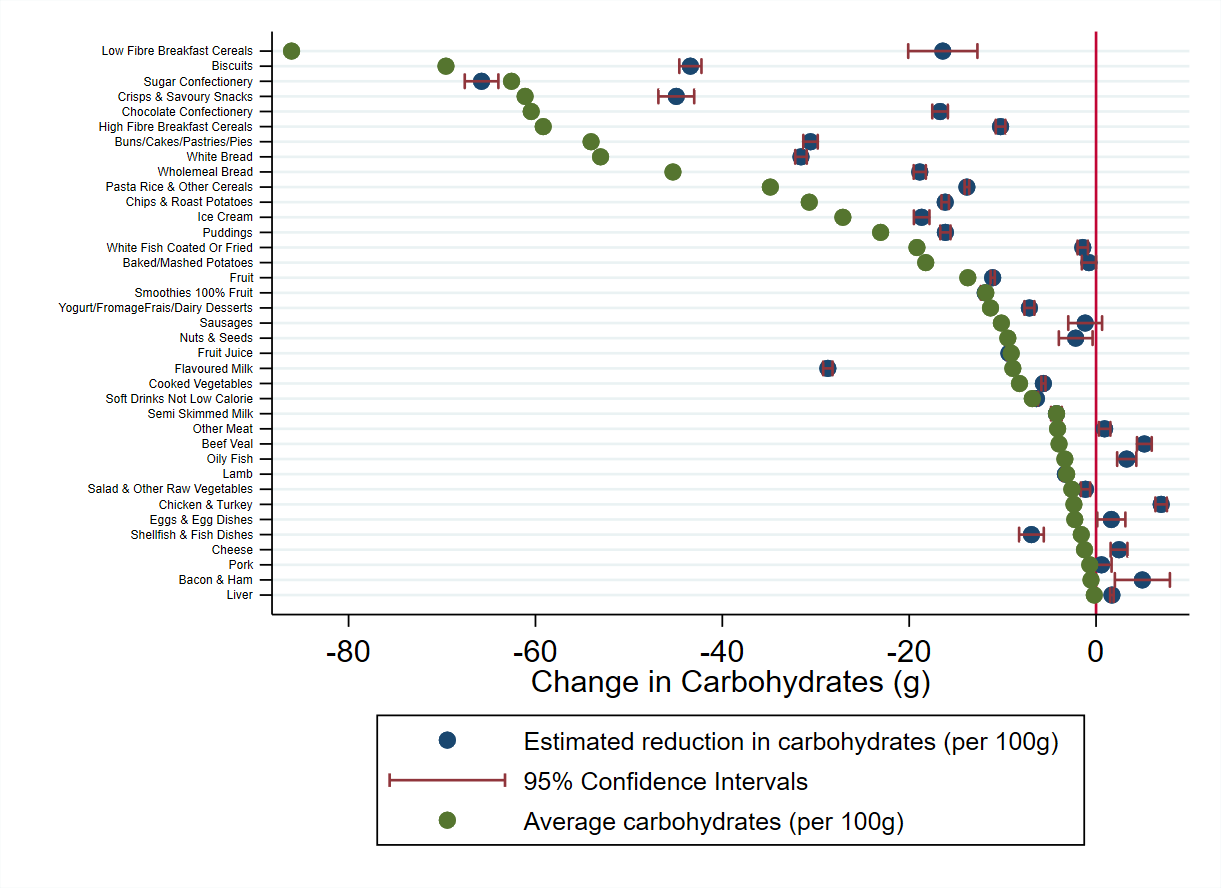


Figure A2 legend: The blue dot illustrates the change in daily carbohydrates (gram) consumption estimated form the UK Biobank data (N=185,611) with precision illustrated by the red confidence intervals. The green dot illustrates the average carbohydrate content (g) for 100g of each food group. The red line at zero illustrates a point where no change in carbohydrate is observed for a 100g reduction in the consumption of each food group. g grams

Figure A3: Estimated change in daily total fat consumed following a reduction in consumption of that food group


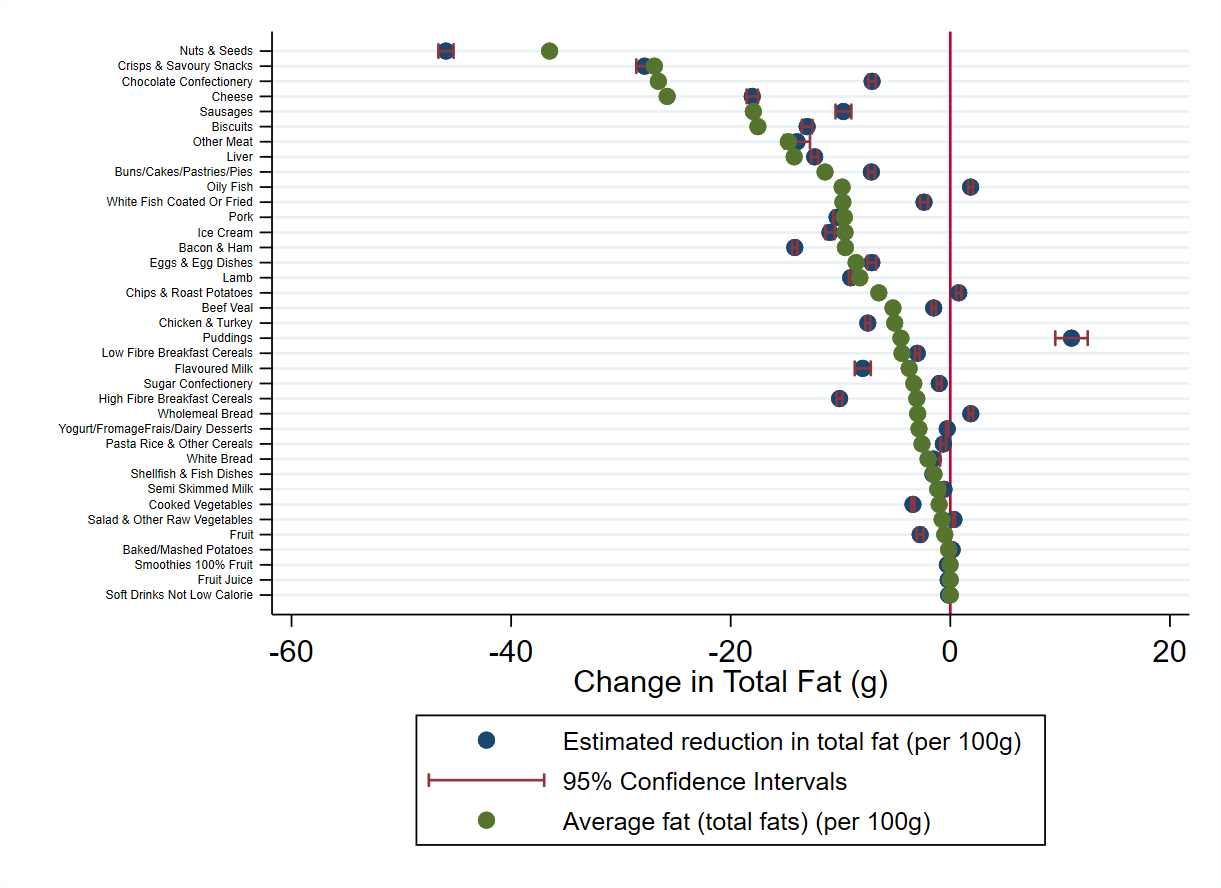


Figure A3 legend: The blue dot illustrates the change in daily total fat (g) consumption estimated form the UK Biobank data (N=185,611) with precision illustrated by the red confidence intervals. The green dot illustrates the average total fat (g) content for 100g of each food group. The red line at zero illustrates a point where no change in total fat is observed for a 100g reduction in the consumption of each food group. g grams.

Figure 4: Estimated change in daily mono unsaturated fat consumed following a reduction in consumption of that food group


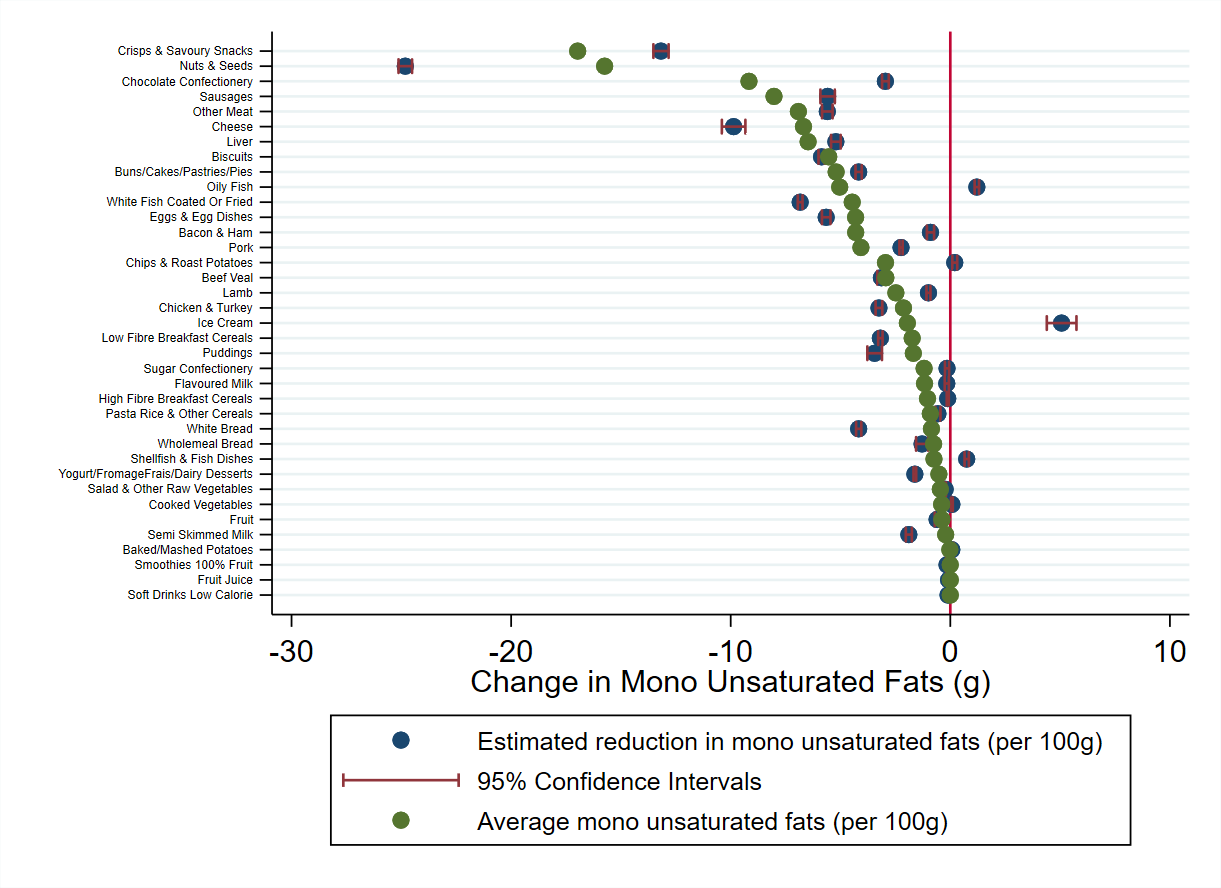


Figure A4 legend: The blue dot illustrates the change in daily mono unsaturated fat (g) consumption estimated form the UK Biobank data (N=185,611) with precision illustrated by the red confidence intervals. The green dot illustrates the average mono unsaturated fat (g) content for 100g of each food group. The red line at zero illustrates a point where no change in mono unsaturated fat is observed for a 100g reduction in the consumption of each food group. g grams.
